# Data in support of 5′AMP-activated protein kinase alpha regulates stress granule biogenesis

**DOI:** 10.1016/j.dib.2015.04.010

**Published:** 2015-05-05

**Authors:** Hicham Mahboubi, Ramla Barisé, Ursula Stochaj

**Affiliations:** Department of Physiology, McGill University, Montréal, Canada

## Abstract

This data article contains insights into the regulation of cytoplasmic stress granules (SGs) by 5′-AMP-activated kinase (AMPK). Our results verify the specific association of AMPK-α2, but not AMPK-α1, with SGs. We also provide validation data for the isoform-specific recruitment of the AMPK-α subunit to SGs using (i) different antibodies and (ii) a distinct cellular model system. In addition, we assess the SG association of the regulatory AMPK β- and γ-subunits. The interpretation of these data and further extensive insights into the regulation of SG biogenesis by AMPK can be found in “5′AMP-activated protein kinase alpha regulates stress granule biogenesis” [1].

Specifications table

**Table:** 

Subject area	Biology
More specific subject area	Stress response, cellular signaling, cell survival
Type of data	Text, figures and graphs
How data was acquired	Immunofluorescence microscopy, quantitative Western blotting
Data format	Analyzed
Experimental factors	Mouse embryonic fibroblasts and human cancer cells (HeLa) treated with different inducers of cellular stress
Experimental features	Drug-treated or transfected cells were processed for immunofluorescence followed by confocal microscopy. Alternatively, cell extracts were prepared and protein content was analyzed by Western blotting
Data source location	McGill University, Montreal, Canada
Data accessibility	All data provided within this article

Value of the data

• Data show isoform-specific association of the catalytic AMPK-α2 subunit with SGs in different cells from distinct organisms.
• The recruitment of the regulatory AMPK-β and AMPK-γ subunits to SGs under different stress conditions is assessed.
• Data on the impact of AMPK knockdown on the abundance of core SG proteins are provided.

## 1 Data, materials and methods

**Fig. 1:**
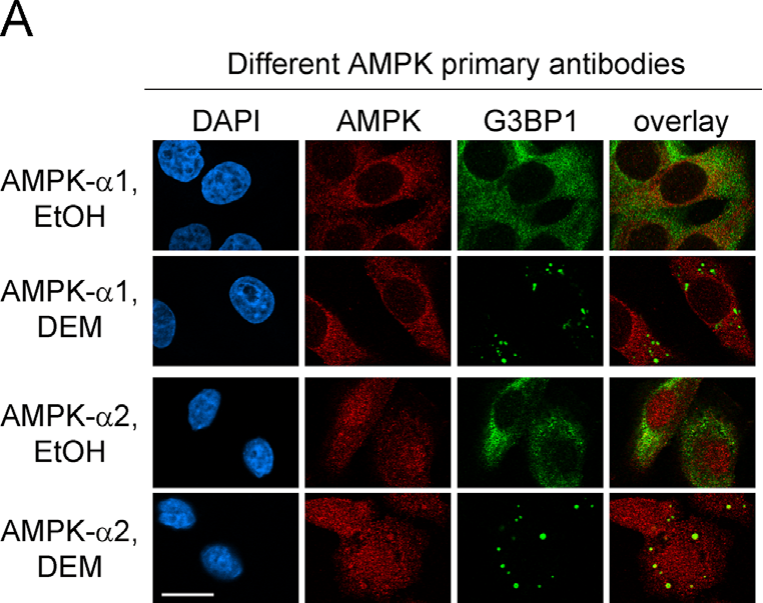
Isoform-specific recruitment of AMPK-α to SGs. The isoform-specific association of the catalytic subunit with SGs was verified in HeLa cells with a different set of primary antibodies against AMPK-α1 or AMPK-α2 (Upstate, see Section 1). Consistent with results in our main paper [1], these unrelated isoform-specific antibodies located AMPK-α2 to DEM-induced SGs, which were demarcated with the SG marker G3BP1. Scale bar is 20 μm.

**Fig. 2:**
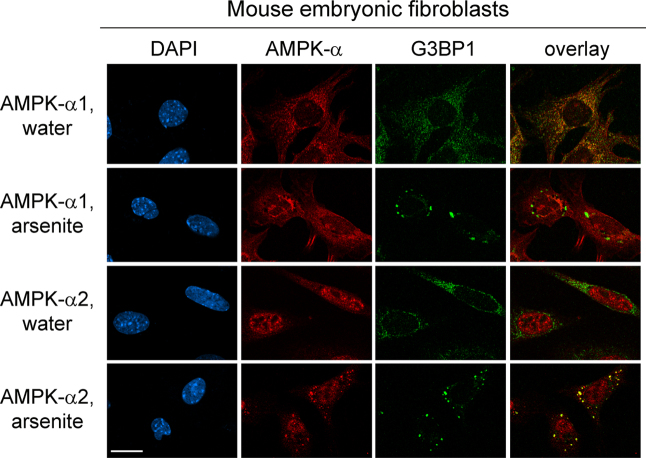
AMPK-α2 locates to SGs in mouse embryonic fibroblasts. Immortalized mouse embryonic fibroblasts were treated with arsenite (Section 1). AMPK-α was detected with isoform-specific antibodies as described for Fig. 2[1]. AMPK-α2, but not AMPK-α1, localized to SGs. Scale bar is 20 μm.

**Fig. 3:**
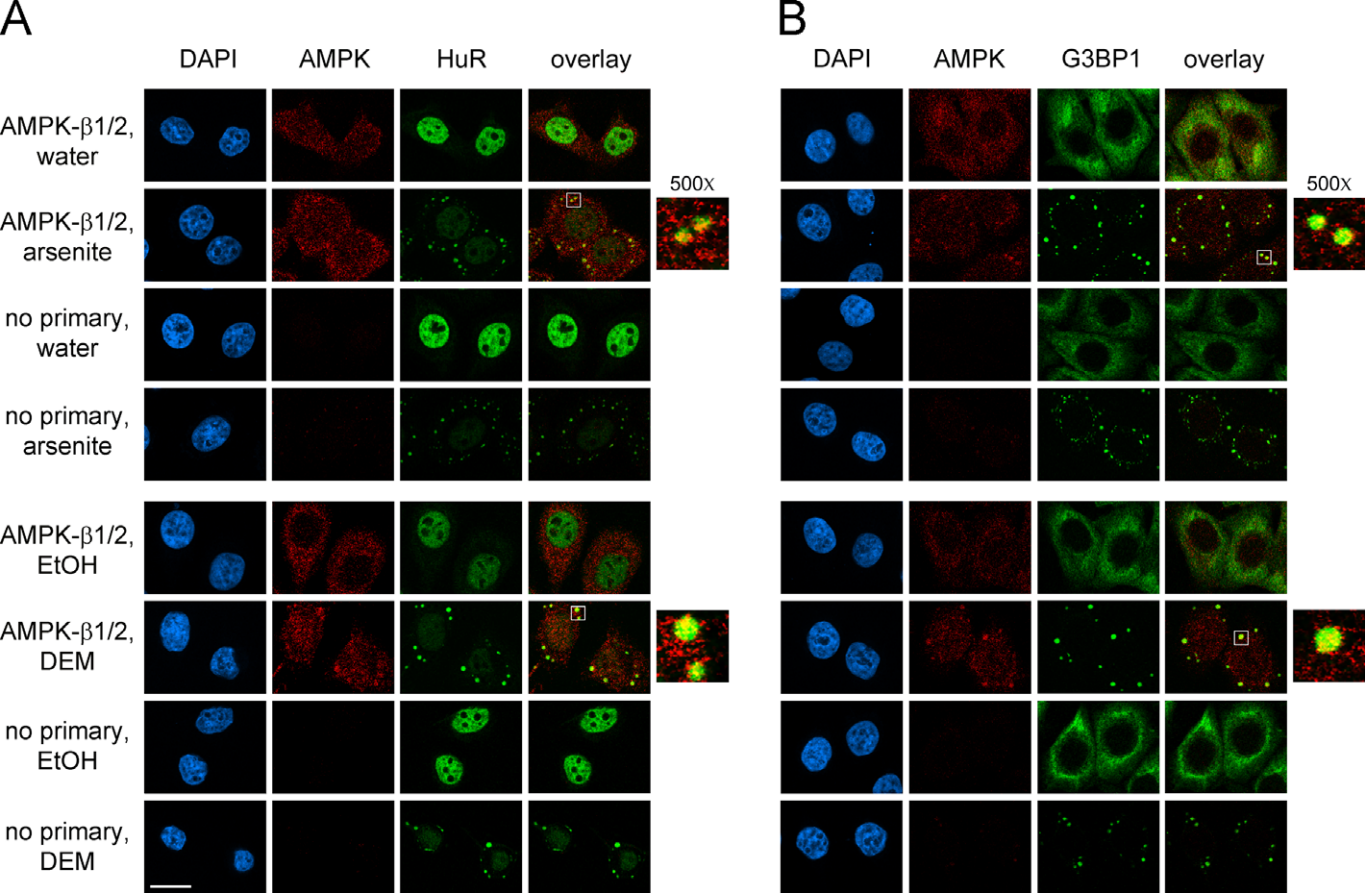
Association of AMPK-β1/2 with SGs. The distribution of AMPK-β1/2 was examined for control and stressed cells. Oxidative stress was induced with arsenite or DEM. SGs were identified with (A) HuR or (B) G3BP1; DNA was stained with DAPI. Control experiments were performed without primary antibodies. For each condition, images were acquired and processed with identical settings. Scale bar is 20 μm.

**Fig. 4:**
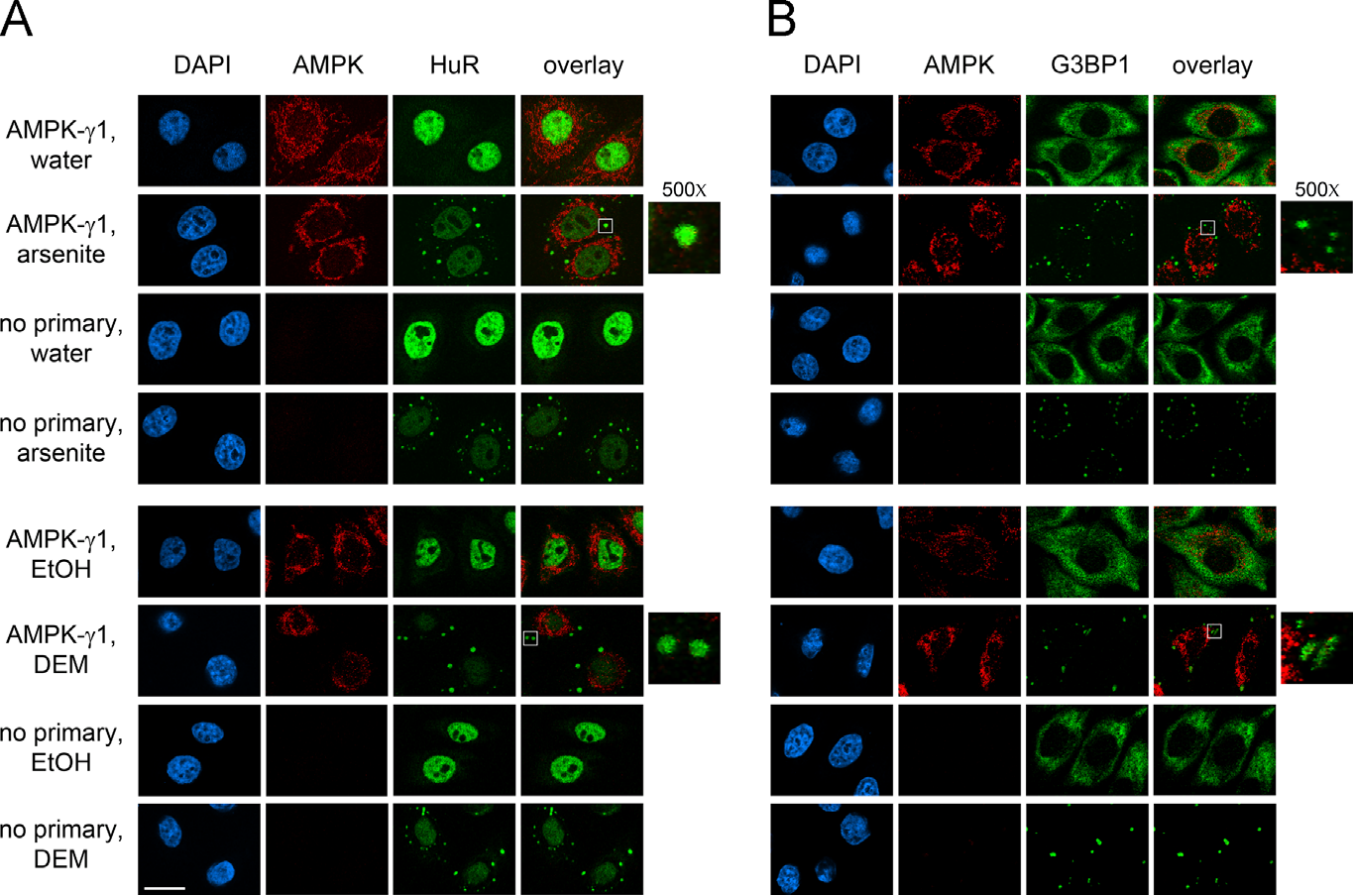
Presence of AMPK-γ1 in SGs. The recruitment to SGs was assessed for AMPK-γ1 as described for Fig. 3. SGs were demarcated with (A) HuR or (B) G3BP1; nuclei were detected with DAPI; scale bar is 20 μm.

**Fig. 5:**
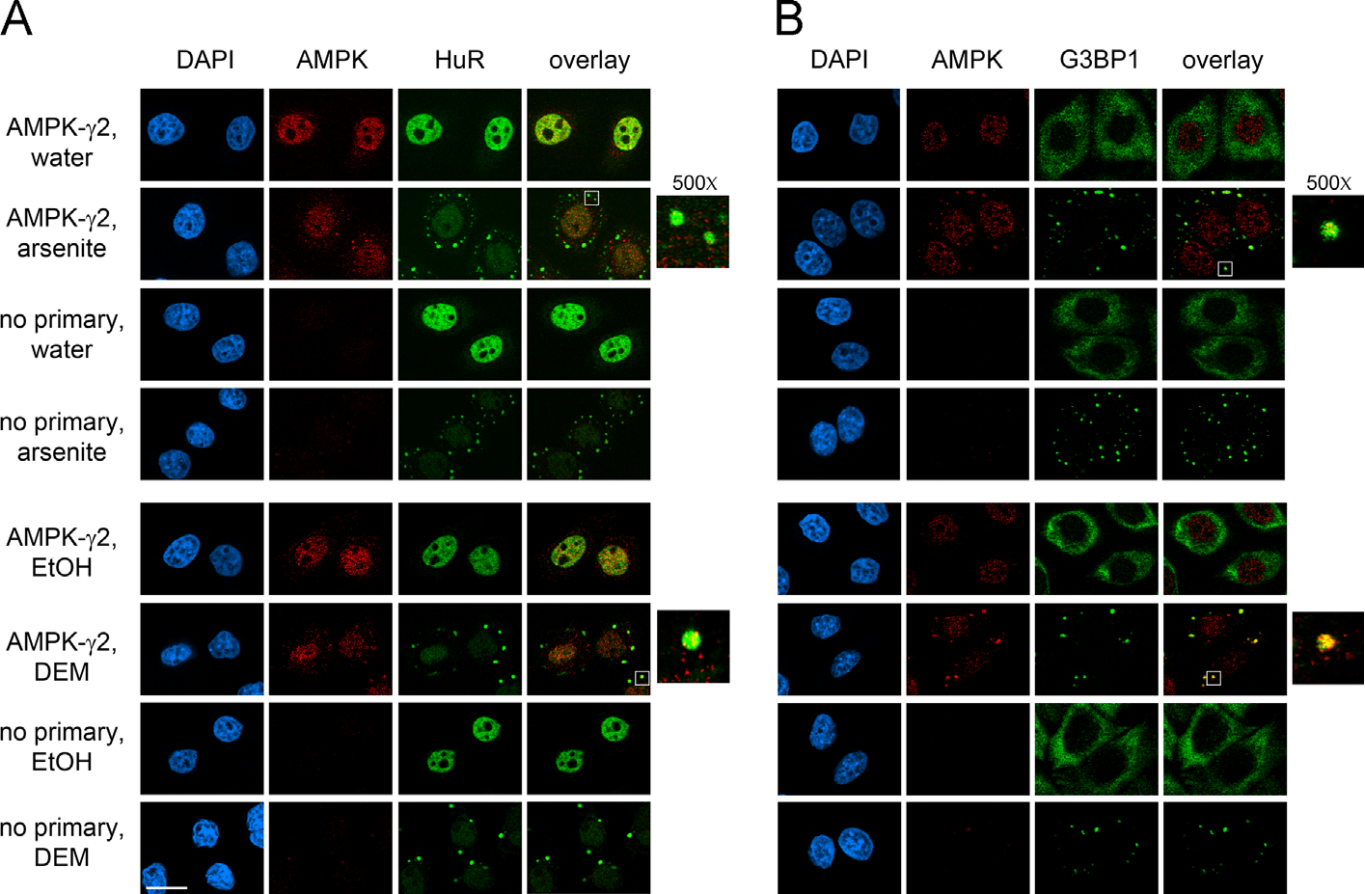
Recruitment of AMPK-γ2 to SGs. The association of AMPK-γ2 with SGs was evaluated as in Fig. 3. SGs were identified with (A) HuR or (B) G3BP1. DNA was stained with DAPI; scale bar is 20 μm.

**Fig. 6:**
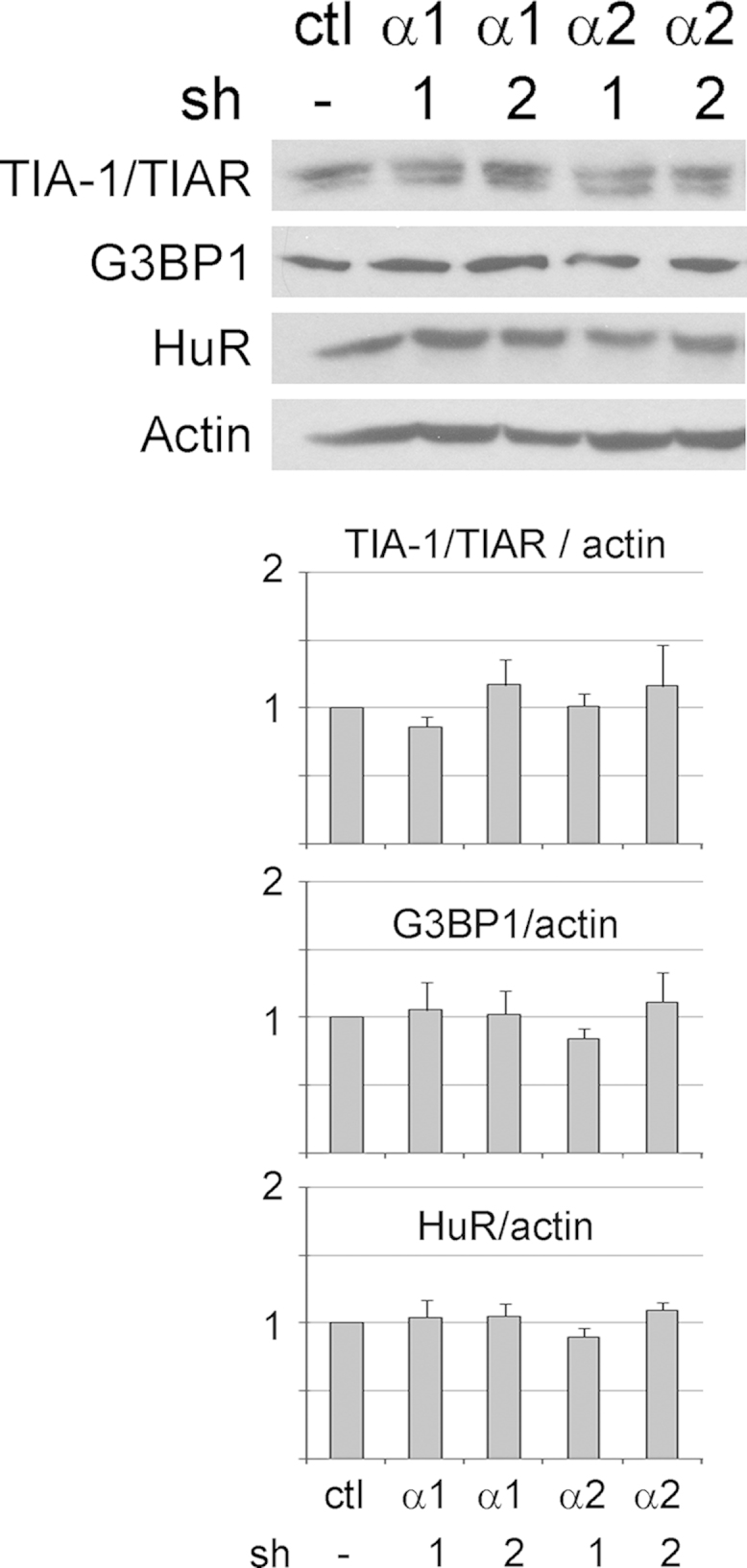
Impact of AMPK-α depletion on the abundance of core SG proteins. Quantitative Western blotting measured the levels of TIA-1/TIAR, G3BP1 or HuR in control and AMPK-α knockdown cells. Actin was used as reference. AMPK-α1 or AMPK-α2 knockdown did not significantly change the concentration of TIA-1/TIAR, G3BP1 or HuR.

AMPK serves as a master metabolic regulator in eukaryotic cells [2]. Moreover, AMPK is also critical to other cellular functions, such as the stress response [3]. The formation of cytoplasmic SGs is one of the hallmark responses to many types of stress [4]. Here, we used different antibodies (Fig. 1) and a distinct cell line from another organism (Fig. 2) to verify the isoform-specific recruitment of the AMPK-α subunit to SGs. In addition to the catalytic α subunits, we assessed the interaction of regulatory β and γ AMPK subunits with SGs upon treatment with arsenite or diethyl maleate (DEM), components that induce oxidative stress (Figs. 3–5). AMPK-α knockdown changed SG parameters [1], and we tested whether this can be linked to the abundance of SG marker proteins TIA-1/TIAR, G3BP1 and HuR. To this end, we quantified the levels of core SG proteins under control and AMPK-α knockdown conditions. The knockdown of AMPK-α1 or α2 did not cause significant changes in the concentration of these three SG marker proteins (Fig. 6).

### 1.1 Cell culture, stress and drug treatment

HeLa cells were grown in Dulbecco׳s modified eagle medium (DMEM) containing antibiotics and 8% Fetal Bovine Serum (FBS). Mouse Embryonic Fibroblasts were cultured in DMEM, supplemented with l-glutamine and 8% FBS. Cultures were maintained in a 37 °C incubator containing 5% CO_2_. To induce oxidative stress, cells were incubated with 0.5 mM sodium arsenite or sterile water (control) for 1 h. Alternatively, HeLa cells were incubated with 2 mM diethyl maleate (DEM) or ethanol (control) for 4 h.

### 1.2 Indirect immunofluorescence

Cells were grown to 70% confluency on poly-lysine coated cover slips. After treatment, immunofluorescent staining was performed following published procedures [5]. Antibody dilutions were: AMPK-α1 and AMPK-α2 (1:1000; Upstate 07-350; 07-363; or 1:2000; Bethyl A300-507A, A300-508A), AMPK-β1/2 (1:400; Cell Signaling ♯4150), AMPK-γ1 (1:200; Cell Signaling ♯4187), AMPK-γ2 (1:200; Cell Signaling ♯2536), HuR (1:2000; sc-5261), G3BP1 (1:1000; BD Biosciences ♯611126). In brief, cells were fixed with 3.7% formaldehyde in phosphate-buffered saline (PBS), permeabilized with 0.1% Triton X-100 in PBS/2 mg/BSA and blocked for 1 h in PBS containing 5% fetal bovine serum (FBS), 0.05% Tween-20 and 1 mM NaN_3_. Samples were incubated overnight with primary antibodies diluted in PBS/FBS/Tween. Purified Alexa Fluor® 488, Alexa Fluor® 647 and Cy3 fluorescently-labeled secondary antibodies were added the following day for 2 h [6]. Nuclei were visualized with 1 µg/ml 4׳,6 Diamidino-2-phenylindole dihydrochloride (DAPI).

### 1.3 Cell extracts

HeLa cell proteins were solubilized in gel sample buffer as described [7]. Samples were incubated at 95 °C for 10 min and vortexed with silica beads to shear DNA. Proteins were precipitated with trichloroacetic acid for 20 min on ice and collected by centrifugation (microfuge, 1 min at 13,000 rpm). Sediments were resuspended in gel sample buffer and analyzed by Western blotting.

### 1.4 Western blotting

Western blotting and ECL followed standard procedures. Primary antibodies were used at the following dilutions: AMPK-α1 and AMPK-α2 (1:2000; Bethyl A300-507A, A300-508A), HuR (1:2000), G3BP1 (1:2000), TIA-1/TIAR (1:1000), and actin (1:100,000). Primary antibodies were detected with HRP-conjugated secondary antibodies.
